# Variations in methyl bromide concentration with distance and time during quarantine fumigation

**DOI:** 10.1007/s10661-021-09154-3

**Published:** 2021-06-08

**Authors:** Min-Goo Park, Young-Seoub Hong, Chung Gyoo Park, Dong-Chul Gu, Hyoung-ho Mo

**Affiliations:** 1 Department of Plant Quarantine, Animal and Plant Quarantine Agency (APQA), Gimcheon, 39660 Republic of Korea; 2 Institute of Agriculture and Life Science (BK21+ Program), Gyeongsang National University, Jinju, 52828 Republic of Korea; 3 Department of Preventive Medicine, College of Medicine, Dong-A University, Busan, Republic of Korea; 4 Heavy Metal Exposure Environmental Health Center, Dong-A University, Busan, Republic of Korea; 5 Department of Public Health, Medical Graduate School, Pusan National University, Busan, Republic of Korea; 6 Gugil Environment and Industrial Hygiene. Co. Ltd, Busan, Republic of Korea

**Keywords:** Declining trends, Fumigation workers, Exposure assessment, Environmental monitoring, Exposure modeling

## Abstract

Methyl bromide (MB) is a highly toxic and ozone-depleting substance and should be replaced. Worker exposure to high MB concentrations during fumigation has been previously reported. However, variations in MB concentration as a function of distance from fumigated objects or of time after degassing have not been reported so far. In this study, air samples were collected at various distances from fumigated objects (oranges, wood in containers, and wood in tarpaulin) during injection and degassing and analyzed via gas chromatography according to the Occupational Safety and Health Agency method. In addition, MB concentrations were directly measured over time using a gas detector during degassing. Non-linear regression analysis of the logarithmically transformed data indicated a clear decrease in MB concentration with distance as well as time. Non-linear regression models were constructed to describe the decrease in MB concentration with distance from the objects and with time during degassing (*P* < 0.05 for all models). The results of this study could aid in establishing appropriate safety guidelines, and hence, in preventing risks related to MB exposure.

**Supplementary Information:**

The online version contains supplementary material available at 10.1007/s10661-021-09154-3.

## Introduction

Methyl bromide (MB) has been used as a disinfectant for removing pests in trade commodities as well as from warehouses and soil since 1930 (Bond, 1984; Fields & White, 2002; Noling & Becker, 1994). Specifically, MB is used in the plant quarantine process because it can easily undergo vaporization and penetrate various materials (Bond, 1984). However, MB was designated as an ozone-depleting substance in the Montreal protocol in 1992 except for quarantine and pre-shipment purposes (UNEP, 2018). Therefore, an alternative chemical for fumigation and disinfecting applications is required, although MB use for quarantine purpose is still not legally restricted in many countries including Republic of Korea (MBTOC, 2014).

MB is highly toxic to humans. The fumigant is absorbed mostly via respiration and causes lung damage, cytotoxicity, genotoxicity, visual disturbance, and various other toxic effects in the human body (Baur et al., 2015; Deschamps & Turpin, 1996; Preisser et al., 2012; Suwanlaong & Phanthumchinda, 2008). Neurological disorders such as nausea, vomiting, and dizziness appear to be the most prevalent effects of MB exposure and are caused by the presence of MB metabolites such as methyl phosphate in cells (Agency for toxic substances and disease registry (ATSDR), 2018; Bulathsinghala & Shaw, 2014). Moreover, the electroencephalogram (EEG) or heart rate variability (HRV) of MB fumigation workers has been shown to decrease to levels indicating functional degradation and weakness of central or autonomic nerves, even though the workers were asymptomatic (Choi et al., 2020; Park et al., 2020). The environmental monitoring of MB has been studied predominantly in the context of soil fumigation (Dimitriou & Tsoukali, 1998; Roosels et al., 1981). Only a few studies have been conducted to assess the potential risks of MB exposure in quarantine areas. In those studies, the MB concentrations in personal air samples were analyzed (Lee & Shin, 2008; Tanaka et al., 1991). The studies were undertaken for risk assessment within various fumigation enclosures, such as containers, or during various fumigation processes, such as dispersion and degassing. However, these studies did not provide a clear explanation as to why workers are exposed to such high MB concentrations and thus how such exposure can be avoided.

In this study, we propose a regression model describing the relationship between MB concentration and the distance from the fumigated objects as well as time from the commencement of degassing. Oranges, wood in containers, and wood in tarpaulin were examined as the fumigated objects. Practical guidelines, in terms of ensuring worker safety, for the distance required between the fumigated objects and the workers as well as the time required between fumigation completion and resumption of subsequent work are provided.

## Materials and methods

### Fumigation work process

The fumigation work process on oranges loaded inside a container was as follows: (1) preparation for application (placing the blower in the container, connecting the MB hose from the MB cylinder to the container, and sealing the door seams with specialized boxing tape for the container), (2) injection of MB for 30 min at a dose of 64 g/m^3^, (3) dispersion for 2 h, (4) degassing for 2 h, and (5) resuming work (e.g., removing fan from the container). The dosage and exposure duration (2 h) has been regulated for disinfecting the harmful insects such as mealy bugs in the Animal and Plant Quarantine Agency (2019). The workers wore gas masks to reduce the exposure to MB while working. The fumigation process on wood stored in containers or tarpaulins was similar to that followed for the fumigation of oranges in the containers. However, the fumigation duration was extended to more than 24 h, instead of just 2 h, in accordance with the regulations stipulated by the Animal and Plant Quarantine Agency (2019). Further, a dose of 33–73 g/m^3^ was applied depending on the temperature of the wood.

### Environmental monitoring of MB in the work area

Environmental monitoring of MB was carried out in two parts during outdoor fumigation at the Busan Port, South Korea, from May 2019 to September 2019: (1) measurement of MB concentration with distance from an object, and (2) measurement of MB concentration over time. The temperature and relative humidity were 21 to 36 ℃ and 34 to 88% during the monitoring (Table S1).

According to US treatment standards, an area outside of 9 m is usually regarded as a safe distance from the fumigated objects (USDA, 2019). Ten meters for degassing was assumed to be the maximum distance for worker safety, and 3 m was designed for injection due to relevant gas tightness of the container or tarpaulin. To assess the MB concentration with distance from objects (oranges in container, wood in container, wood in tarpaulin), air samples were collected at distances of 0, 1, and 3 m or 0, 1, 3, 6, and 10 m, in parallel to injection or degassing direction, from points of fumigation enclosure, such as the container door, depending on the injection or degassing conditions. Each measurement was repeated three times independently on 1 container or tarpaulin.

Air samples with flow rates of 0.1 L/min were obtained with a portable low-flow sampler (Gilian, USA) connected to an activated carbon tube (400 mg/200 mg, SKC, USA). The samples were collected for 2 h, which was considered to be the maximum duration of MB exposure during each injection and degassing event. The collected samples were placed in a 2-mL vial, with 1 mL of CS_2_ containing octane used as an internal standard. The caps were immediately closed, and the vials were subjected to vibration for 30 min to promote desorption. The MB concentrations in the samples were analyzed using gas chromatography (Agilent 6890 series; Agilent Technologies, CA, USA) with a flame ionization detector (FID). The calibration curve was drawn with seven working standards over the range of 17.3 to 1384.0 μg/mL of MB in CS_2_ solution spiked with known quantity of MB. The limit of detection for MB was calculated to be 1.44 μg per sample based on the calibration, which is equivalent to approximately 0.36 µL/L of MB, less than the threshold limit value (TLV) of 1 ppm, trapped at an airflow rate of 0.1 L/min for 2 h. The conditions of gas chromatography are shown in Table S2. Technical details of the analyses are described by the US Occupational Safety and Health Agency (OSHA, 2019).

To determine the MB concentration with time after the initiation of degassing, the MB concentrations were directly measured using a gas detector (Gas tiger 2000; Shenzhen Wandi Technology Co. Ltd, China) for 130 s at a distance of 10 m from each fumigated object at 0, 1, and 2 h from the commencement of degassing. The concentration was measured at 10-s intervals for up to 130 s (Fig. S1). The device was equipped with a photoionization detector, which was certified with a detection error of 3% for MB by Shenzhen Wandi Technology Co. Ltd. in February 2019. The portable detector was selected for the assessment with time because the MB concentration needs to be monitored over a wide distance (0–10 m) and for a short duration (130 s) at certain elapsed times during degassing (2 h). The MB concentration as a function of time was monitored only during degassing, which is more risky in terms of worker safety due to higher MB concentrations at this stage (Tanaka et al., 1991). The wind flow was checked at distances of 0, 1, and 3 m or 0, 1, 3, 6, and 10 m from the objects, both for injection and degassing conditions. The flow was directly measured using air velocity meter (TSI 9545A, TSI Co. Ltd, USA) at 1-h intervals for 2 h; it took ~30 s for one reading. As mentioned before, each measurement was repeated three times independently.

### Statistical analysis

The measured MB concentrations are presented as the mean ± standard deviation (SD) (Table 1). The trend in MB concentration with distance or time was analyzed using non-linear regression by means of an exponential decay function (Eq. 1) with two model parameters. The parameters were estimated via dynamic curve fitting in SigmaPlot, version 12.0 (Systat Software, San Jose, CA, USA). Each MB concentration was transformed to a logarithmic scale to fulfill the normal distribution assumptions required for parametric statistical analysis. An exponential decay model is commonly used for gas concentration, and all the coefficients of determination were higher than 0.80.

**Table 1:** Mean MB concentrations (µL/L) at various distances from the fumigated objects during both injection and degassing and at various elapsed times after the start of degassing

		Orange in container		Woods in container		Woods in tarpaulin	
		Injection	Degassing	Injection	Degassing	Injection	Degassing
Distance (m)	0	1238.2 ± 393.6	1296.4 ± 410.1	612.3 ± 33.2	494.8 ± 361.9	1289.6 ± 896.9	337.2 ± 276.3
	1	96.4 ± 83.8	267.3 ± 87.8	79.9 ± 57.4	78.7 ± 73.5	44.9 ± 40.0	27.7 ± 28.8
	3	11.3 ± 0.6	34.6 ± 25.5	5.0 ± 8.6	21.6 ± 15.8	N.D	5.4 ± 9.4
	6	–	6.1 ± 5.3	–	1.9 ± 3.3	–	N.D
	10	–	N.D	–	N.D	–	N.D
Time (h)	0	–	544.1 ± 462.1	–	668.1 ± 599.4	–	497.1 ± 315.3
	1	–	86.6 ± 79.1	–	45.9 ± 40.2	–	77.8 ± 46.3
	2	–	3.7 ± 3.1	–	3.0 ± 2.7	–	4.7 ± 1.3

Each measurement was conducted three times independently. Data are expressed as mean ± SD of the three replicate experiments

*N.D.* below the detection limit of 0.36 ppm

1 $$y={ae}^{-bx}$$

Here, y is the MB concentration in the logarithmic scale, x is the elapsed time (h) or distance (m) from the fumigated object, and a and b are the estimated parameters.

## Results and discussion

**Fig. 1:**
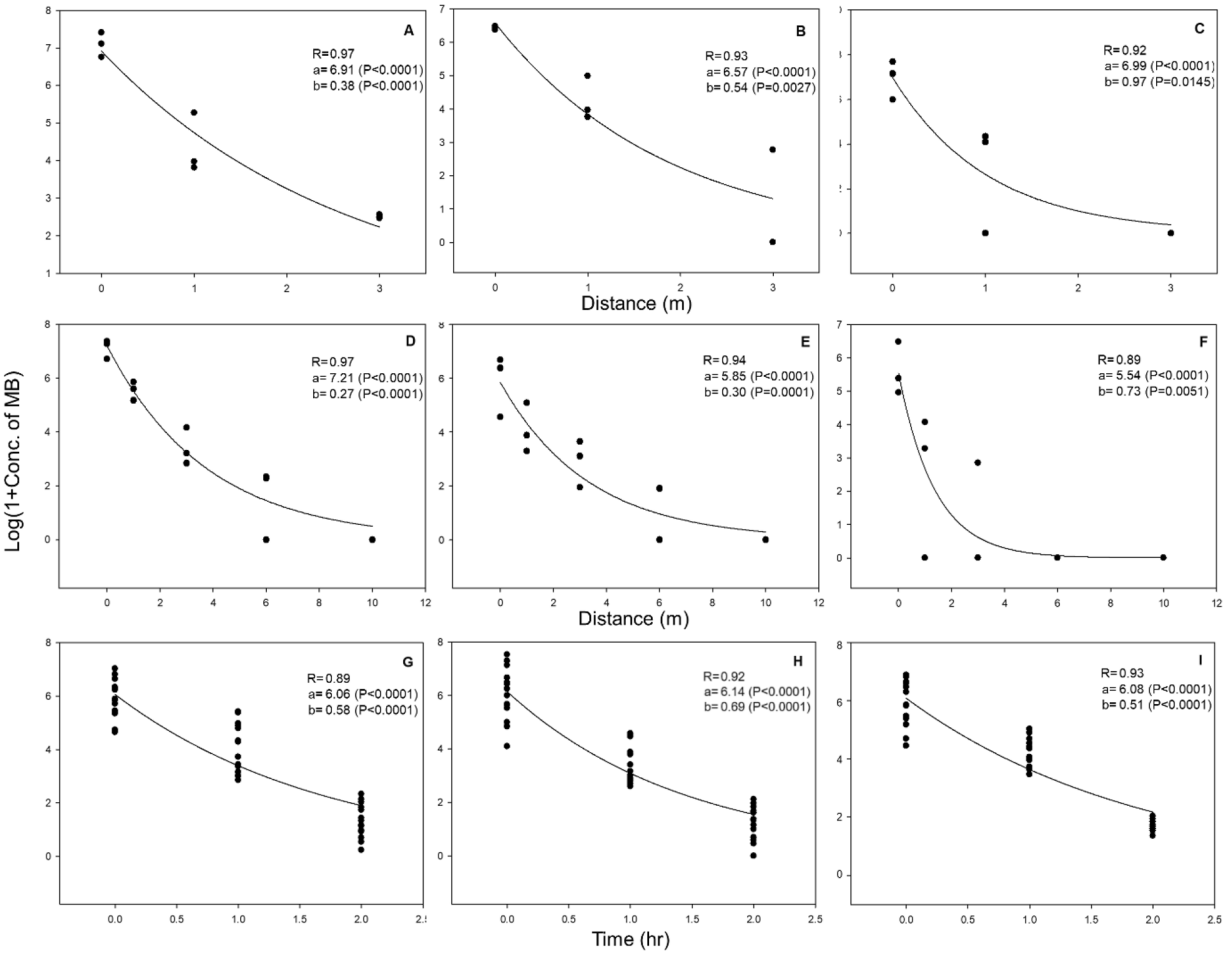
MB concentration measured at 0, 1, and 3 m from the objects (**A** orange in container, **B** wood in container, **C** wood in tarpaulin) at the start of MB gas injection and measured at 0, 1, 3, 6, and 10 m from the objects (**D** orange in container, **E** wood in container, **F** wood in tarpaulin) at the start of MB gas exhaust, and measured at 0, 1, 2 h after the start of MB gas exhaust (**G** orange in container, **H** wood in container, **I** wood in tarpaulin). The data are expressed in the common log-scale to fulfill the normal distribution assumptions for statistical analysis

The MB concentration decreased with distance during both the injection and degassing processes for all three treated commodities, namely, oranges in containers, woods in containers, and woods in tarpaulin (Table 1). In addition, the concentrations decreased with time during degassing for all commodities (Table 1). As shown in Fig. 1, the fitting curves for the MB concentrations obtained using the exponential decay model describe the relationships well (*P* < 0.05 for all models). The MB concentrations decreased non-linearly with distance from the oranges during injection (*y* = 6.91e^−0.38*x*^) as well as during degassing (*y* = 7.21e^−0.27*x*^). Similar relationships were observed during the injection and degassing steps for wood in the container (*y* = 6.57e^−0.54×^ and *y* = 5.85e^−0.30*x*^, respectively) and wood in tarpaulin (*y* = 6.99e^−0.97×^ and *y* = 5.54e^−0.73*x*^, respectively). Further, the MB concentration decreased with time during the degassing step in the fumigation of oranges (*y* = 6.06e^−0.58*x*^), wood in containers (*y* = 6.14e^−0.69*x*^), and wood in tarpaulin (*y* = 6.08e^−0.51*x*^).

In 1981, the American Conference of Governmental Industrial Hygienists (ACGIH) set the TLV for MB at 5 ppm (v/v) for an 8-h time-weighted average (TWA). However, this value was revised to 1 ppm in 1997 (ACGIH, 2005; Lee & Shin, 2008). The OSHA (2019) has set a standard of 20 ppm (ceiling), while NIOSH describes MB as a potential carcinogen and recommends minimizing exposure as much as possible (CDC, 2011). Lee and Shin (2008) reported that the MB concentrations in the personal air samples of 4 out of 27 fumigators (15%) exceeded the TLV of 1 ppm. In addition, the mean and maximum concentrations of MB in 63 short-term air samples were reported to be 17.7 and 340.7 ppm, respectively. Tanaka et al. (1991) showed that 67 out of 107 fumigators (63%) were engaged in degassing processes in which the MB concentration exceeded the TLV of 5 ppm. MB concentrations of 74.6, 31.8, and 19.8 ppm were measured during the degassing process in log yards, ships, and warehouses, respectively.

In the current study, the mean MB concentration 3 m away from the fumigated objects during both the injection and degassing processes was lower than the OSHA permissible exposure limit (PEL; ceiling of 20 ppm), except in the cases of oranges and wood in container (34.6 and 21.6 ppm) during the degassing process (Table 1). The mean concentration at a 1-m distance from the objects was less than 267.3 ppm. These results were similar to those of short-term air samples or personal readings obtained at degassing sites in previous studies (Lee & Shin, 2008; Tanaka et al., 1991). However, the mean MB concentrations during the injection and degassing processes were 1238.2 and 1296.4 ppm, 612.3 and 494.8 ppm, and 1289.6 and 337.2 ppm in the proximities of the oranges, wood in containers, and wood in tarpaulin, respectively (Table 1). It is noteworthy that the concentrations measured were approximately 60 times the OSHA (2019) PEL (ceiling of 20 ppm) in the proximity of the fumigated oranges, both during the injection and degassing processes.

The mean concentrations during degassing in the cases of wood in container and tarpaulin were 668.1 and 497.1 ppm at 0 h, 45.9 and 77.8 ppm at 1 h, and 3.0 and 4.7 ppm at 2 h, respectively, as measured with the gas detector (Table 1). These values are quite similar to the mean values (494.8 and 337.3 ppm at 0 m, 78.7 and 27.7 ppm at 1 m, 21.6 and 5.4 ppm at 3 m, 1.9 and 0 ppm at 6 m from wood), as assessed by the standard OSHA method (Table 1). We derived the relationship between MB concentration and time for all three cases only with the values obtained from a gas detector, although the measurements with the gas detector were not as accurate as that with a gas chromatograph.

In previous studies, even though most of the workers wore masks, it was reported that their health was negatively affected owing to exposure to MB (Choi et al., 2020; Park et al., 2020). However, it is not known whether the workers were actually exposed to harmful concentrations, since the concentrations in the workplace were not provided in the studies. In this study, we provide evidence that the concentration in the work area is at a sufficiently high level to cause harm to the health of workers. In addition, this data clarifies the cause for exposure to high concentrations (i.e., working too close to the object or resuming work before MB is sufficiently exhausted from the work area).

The decreasing MB concentration trends in Fig. 1 were statistically significant (*P* < 0.05 for all models). In particular, clear non-linear regression models describing the relationships between MB concentration and key variables have been provided. This approach is different from that employed in previous studies, in which only the effects of workers’ exposure to airborne MB were studied (Lee & Shin, 2008; Tanaka et al., 1991). The model provided here may be utilized to quantitatively estimate the MB concentration as a function of distance from the objects or elapsed time after the start of degassing. If the concentration estimated by this non-linear regression model in the area is higher than the OSHA PEL or TLV-TWA (20 or 1 ppm, respectively), the corresponding time or distance can be categorized as high risk. Therefore, the workers may be prevented from exposure with some recommended guidelines. With 20 ppm defined as the safe MB concentration level in the work system at the Busan port, during injection, workers should maintain at least 4.35, 2.97, and 1.72 m from the oranges in containers, wood in containers, or wood in tarpaulin, respectively, and in the case of degassing, at least 6.28, 4.96, and 1.96 m, respectively. Further, work should be resumed only at 2.99 h or longer after the start of degassing. This value was estimated by applying the values of the coefficients *a* and *b* in Eq. 1 in Fig. 1.

There are several limitations to the methods used in this study. As this study was conducted in ports where access is strictly restricted, many difficulties were encountered when selecting appropriate objects and determining a place for conducting the measurements. The sample sizes of the areas were often insufficient. There were large differences in ambient conditions during repetitions of the experiments, such as wind flow variations (Table S3). Further, the MB concentration as a function of time during degassing was assessed using a portable gas detector. Therefore, in future studies, the data obtained from the gas detector should be supplemented with evaluation using the standard OSHA method (2019).

Nevertheless, a clear decreasing trend of MB concentration with an increase in distance and time is shown in this study, which will be useful to assess and prevent risks related to MB exposure. Moreover, it is expected that workers will more actively follow safety guidelines such as wearing gas masks, ensuring sufficient gas exhaust, and keeping away from the safety line with these guidelines.

## Conclusion

The MB concentrations in work areas were assessed at various distances from fumigated objects (oranges in containers, wood in containers, and wood in tarpaulin) and at various elapsed times after the commencement of degassing. Non-linear regression models with statistical significance were constructed to describe the relationship between MB concentration during fumigation and variables such as distance from the object and time since the commencement of degassing (*P* < 0.05 for all models). The MB concentrations decreased exponentially with distance and time for all the fumigated objects studied. Based on the clear decreasing trend in MB concentration with distance or time, appropriate safety guidelines can be established to prevent risks related to MB exposure.

Furthermore, this trend only revealed in oranges and woods will provide a basis for expanding the investigation into more items treated with MB for the worker’s safety and will encourage MB to be replaced or restricted in order to reduce its “hidden risk.”

## Supplementary Information

Below is the link to the electronic supplementary material.

Supplementary file1 (DOC 131 KB)

## Data Availability

All data supporting the findings of this study are available from the corresponding authors upon reasonable request.

## References

[CR1] ACGIH. (2005). *2005 TLVs and BEIs*. *ACGIH worldwide*. Cincinnati, OH.

[CR2] Agency for toxic substances and disease registry (ATSDR). (2018). *Toxicological profile for bromomethane*. U.S. Department of health and human services. 37023234

[CR3] Animal and Plant Quarantine Agency. (2019). Regulations for phytosanitary treatment of import and export plant. http://www.qia.go.kr/bbs/lawAnn/viewLawWebAction.do?id=167361&type=0. Accessed 2 March 2020

[CR4] Baur X, Budnik LT, Zhao Z, Bratveit M, Djurhuus R, Verschoor L (2015). Health risks in international container and bulk cargo transport due to volatile toxic compounds. Journal of Occupational Medicine and Toxicology.

[CR5] Bond, E. J. (1984). *Manual of fumigation for insect control* (second.). FAO. http://www.fao.org/3/x5042e/x5042E00.htm

[CR6] Bulathsinghala AT, Shaw IC (2014). The toxic chemistry of methyl bromide. Human & Experimental Toxicology.

[CR7] CDC (2011). Illness associated with exposure to methyl bromide-fumigated produce-California, 2010. Morbidity and Mortality Weekly Report.

[CR8] Choi, J., Hong, Y. S., Cha, W., Mo, H., Park, M. G. (2020). Heart rate variability analysis in workers exposed to methyl bromide as a quarantine treatment. *Journal of Occupational & Environmental Medicine, Publish Ah*, 32–38. 10.1097/jom.0000000000002083 10.1097/JOM.0000000000002083 PMC7773168 33177473

[CR9] Deschamps FJ, Turpin JC (1996). Methyl bromide intoxication during grain store fumigation. Occupational Medicine.

[CR10] Dimitriou A, Tsoukali H (1998). Personal and environmental air sampling of methyl bromide during experimental greenhouse fumigation. Journal of Environmental Science and Health - Part B Pesticides, Food Contaminants, and Agricultural Wastes.

[CR11] Fields PG, White NDG (2002). Alternatives to methyl bromide treatments for stored-product. Annual Review of Entomology.

[CR12] Lee HS, Shin YC (2008). Workers’ exposure to airborne methyl bromide in the exporting/importing plants and products quarantine company. Journal of Korean Society Cccupational Environmental Hygiene.

[CR13] MBTOC. (2014). *2014 report of the methyl bromide technical options committee*. United Nations Environment Programme.

[CR14] Noling JW, Becker JO (1994). The challenge of research and extension to define and implement alternatives to methyl bromide. Journal of Nematology.

[CR15] OSHA. (2019). Permissible exposure limits - annotated tables. https://www.osha.gov/dsg/annotated-pels/tablez-1.html. Accessed 16 January 2020

[CR16] Park M-G, Choi J, Hong Y-S, Park CG, Kim B-G, Lee S-Y (2020). Negative effect of methyl bromide fumigation work on the central nervous system. PLoS ONE.

[CR17] Preisser AM, Budnik LT, Baur X (2012). Health effects due to fumigated freight containers and goods: how to detect, how to act. International Maritime Health.

[CR18] Roosels D, Van Den Oever R, Lahaye D (1981). Dangerous concentrations of methyl bromide used as a fumigant in Belgian greenhouses. International Archives of Occupational and Environmental Health.

[CR19] Suwanlaong K, Phanthumchinda K (2008). Neurological manifestation of methyl bromide intoxication. Journal of the Medical Association of Thailand.

[CR20] Tanaka S, Abuku S, Seki Y, Imamiya S (1991). Evaluation of methyl bromide exposure on the plant quarantine fumigators by environmental and biological monitoring. Industrial Health.

[CR21] UNEP. (2018). *Handbook for the Montreal Protocol on Substances that Deplete the Ozone Layer* (twelfth ed.). United Nations Environment Programme.

[CR22] USDA. (2019). *Treatment Manual*. https://www.aphis.usda.gov/import_export/plants/manuals/ports/downloads/treatment.pdf. Accessed 1 June 2020

