# Correction to: Variations in methyl bromide concentration with distance and time during quarantine fumigation

**DOI:** 10.1007/s10661-021-09246-0

**Published:** 2021-08-13

**Authors:** Min-Goo Park, Hyoung-ho Mo, Young-Seoub Hong, Chung Gyoo Park, Dong-Chul Gu

**Affiliations:** 1 Department of Plant Quarantine, Animal and Plant Quarantine Agency (APQA), Gimcheon, 39660 Republic of Korea; 2 Institute of Agriculture and Life Science (BK21+Program), Gyeongsang National University, Jinju, 52828 Republic of Korea; 3 Department of Preventive Medicine, College of Medicine, Dong-A University, Busan, Republic of Korea; 4 Heavy Metal Exposure Environmental Health Center, Dong-A University, Busan, Republic of Korea; 5 Department of Public Health, Medical Graduate School, Pusan National University, Busan, Republic of Korea; 6 Gugil Environment and Industrial Hygiene. Co. Ltd, Busan, Republic of Korea

## Correction to: Environ Monit Assess (2021) 193:397 https://doi.org/10.1007/s10661-021–09154-3

    The article Variations in methyl bromide concentration with distance and time during quarantine fumigation, written by Min-Goo Park, Young-Seoub Hong, Chung Gyoo Park, Dong-Chul Gu and Hyoung-ho Mo, was originally published Online First without Open Access. After publication in volume 193, issue 7, article ID 397 the author decided to opt for Open Choice and to make the article an Open Access publication. Therefore, the copyright of the article has been changed to © The Author(s) 2021 and the article is forthwith distributed under the terms of the Creative Commons Attribution-NonCommercial-ShareAlike 4.0 International License, which permits any non-commercial use, sharing, adaptation, distribution and reproduction in any medium or format, as long as you give appropriate credit to the original author(s) and the source, provide a link to the Creative Commons licence, and indicate if changes were made. If you remix, transform, or build upon this article or a part thereof, you must distribute your contributions under the same licence as the original. The images or other third party material in this article are included in the article’s Creative Commons licence, unless indicated otherwise in a credit line to the material. If material is not included in the article’s Creative Commons licence and your intended use is not permitted by statutory regulation or exceeds the permitted use, you will need to obtain permission directly from the copyright holder. To view a copy of this licence, visit https://creativecommons.org/licenses/by-nc-sa/4.0/.

