# Supplementary material for: Variations in methyl bromide concentration with distance and time during quarantine fumigation
Source: Environ Monit Assess. 2021 Jun 8;193(7):397. doi: 10.1007/s10661-021-09154-3 (PMC8263420; doi:10.1007/s10661-021-09154-3)
Supplement: Supplementary file 1 — Supplementary file1 (DOC 131 KB) [file 10661_2021_9154_MOESM1_ESM.doc]

**Variations in methyl bromide concentration with distance and time during quarantine fumigation**

**Supplementary materials**


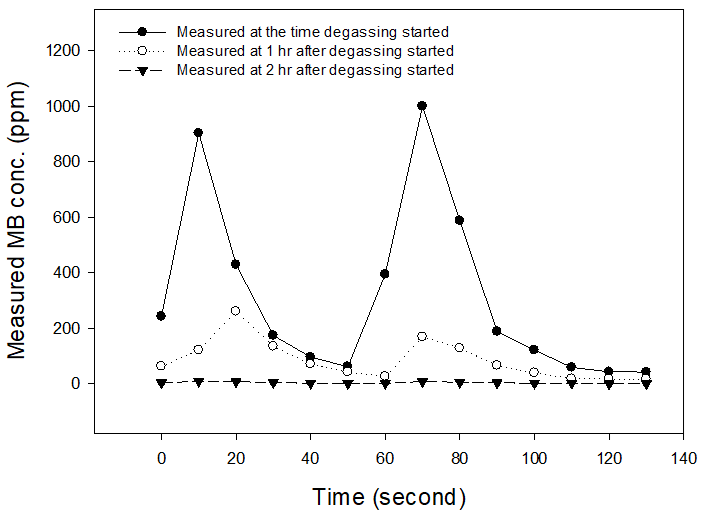


Figure S1. MB concentration measured using the gas detector (Gas tiger 2000, China) at 0, 1, and 2 h after the commencement of degassing for one orange fumigation. MB concentration was high (↓) in the proximity of the objects as it was measured while walking to a distance of 10 m from the proximity of the fumigated objects.

Table S1. The range of temperature (℃) and relative humidity (%) at the time/place of the monitoring

| Object | Factor | Replication 1 | Replication 2 | Replication 3 |
| --- | --- | --- | --- | --- |
| Oranges in container | Temperature | 22–25 | 23–26 | 23–27 |
|  | Humidity | 35–47 | 37–41 | 34–44 |
| Woods in container | Temperature | 21–26 | 23–26 | 21–26 |
|  | Humidity | 36–59 | 65–69 | 62–75 |
| Woods in tarpaulin | Temperature | 28–36 | 27–36 | 25–28 |
|  | Humidity | 49–66 | 57–66 | 81–88 |

Table S2. Gas chromatographic conditions for analyzing methyl bromide

| Parameter | Analytical conditions |
| --- | --- |
| Instrument  Column  Temperature  Oven  Injector  Detector  Injection Volume  Carrier Gas  Solvent | GC, HP-6890  HP-1 MS (30 m × 0.25 mm × 0.5 μm)  35-130 ℃  250 ℃  290 ℃  1 μL  N2 30 /min  CS_2_ |

Table S3. Mean wind flow (m/s) at various distances from the fumigated objects during both injection and degassing

| Object | Timing | Distance (m) | Replication  1 | Replication 2 | Replication  3 |
| --- | --- | --- | --- | --- | --- |
| Oranges in container | Injection | 0 | 0.60 | 0.50 | 0.35 |
|  |  | 1 | 1.05 | 0.55 | 0.60 |
|  |  | 3 | 0.95 | 1.05 | 0.75 |
|  | Degassing | 0 | 0.70 | 0.70 | 0.95 |
|  |  | 1 | 0.65 | 0.60 | 0.65 |
|  |  | 3 | 0.95 | 1.05 | 0.60 |
|  |  | 6 | 0.70 | 1.25 | 1.00 |
|  |  | 10 | 1.10 | 1.10 | 1.15 |
| Woods in container | Injection | 0 | 0.85 | 0.90 | 1.55 |
|  |  | 1 | 1.25 | 0.80 | 0.95 |
|  |  | 3 | 1.00 | 1.00 | 1.30 |
|  | Degassing | 0 | 0.70 | 0.50 | 1.00 |
|  |  | 1 | 1.25 | 0.45 | 1.65 |
|  |  | 3 | 1.00 | 0.60 | 1.40 |
|  |  | 6 | 1.50 | 0.75 | 1.85 |
|  |  | 10 | 1.15 | 0.60 | 1.85 |
| Woods in tarpaulin | Injection | 0 | 0.85 | 1.80 | 1.65 |
|  |  | 1 | 1.20 | 2.20 | 1.50 |
|  |  | 3 | 0.55 | 2.00 | 0.30 |
|  | Degassing | 0 | 0.70 | 1.35 | 2.20 |
|  |  | 1 | 0.35 | 1.35 | 0.80 |
|  |  | 3 | 0.80 | 1.15 | 0.90 |
|  |  | 6 | 1.00 | 0.90 | 0.35 |
|  |  | 10 | 0.55 | 0.55 | 0.50 |

Data are expressed as the mean of three readings at 0, 1, 2 h from the start of each injection or degassing event.
